# Efficacy of family mediation and the role of family violence: study protocol

**DOI:** 10.1186/1471-2458-14-57

**Published:** 2014-01-21

**Authors:** Helen Cleak, Margot Schofield, Andrew Bickerdike

**Affiliations:** 1 Department of Social Work and Social Policy, La Trobe University, Melbourne, Victoria 3086, Australia; 2 School of Public Health and Human Biosciences, La Trobe University, Melbourne, Victoria 3086, Australia; 3 Relationships Australia Victoria, 46 Princess Street, Kew, Victoria 3101, Australia

**Keywords:** Family violence, Family mediation, Coercive behaviour, Conflict tactics scale, Financial control, Separated couples

## Abstract

**Background:**

Family law reforms in Australia require separated parents in dispute to attempt mandatory family dispute resolution (FDR) in community-based family services before court attendance. However, there are concerns about such services when clients present with a history of high conflict and family violence. This study protocol describes a longitudinal study of couples presenting for family mediation services. The study aims to describe the profile of family mediation clients, including type of family violence, and determine the impact of violence profiles on FDR processes and outcomes, such as the type and durability of shared parenting arrangements and clients’ satisfaction with mediated agreements.

**Methods:**

A mixed method, naturalistic longitudinal design is used. The sampling frame is clients presenting at nine family mediation centres across metropolitan, outer suburban, and regional/rural sites in Victoria, Australia. Data are collected at pre-test, completion of mediation, and six months later. Self-administered surveys are administered at the three time points, and a telephone interview at the final post-test. The key study variable is family violence. Key outcome measures are changes in the type and level of acrimony and violent behaviours, the relationship between violence and mediated agreements, the durability of agreements over six months, and client satisfaction with mediation.

**Discussion:**

Family violence is a major risk to the physical and mental health of women and children. This study will inform debates about the role of family violence and how to manage it in the family mediation context. It will also inform decision-making about mediation practices by better understanding how mediation impacts on parenting agreements, and the implications for children, especially in the context of family violence.

## Background

Marriage is a major protective factor for adult wellbeing [1]. Conversely, major conflict in the couple relationship is associated with negative mental health and wellbeing outcomes [2]. For instance, couple conflict predicts a higher incidence of mental disorders in adults, as well as negative social outcomes [2]. The quality of the couple relationship is thus a critical factor in adult mental health and wellbeing.

Extending from the couple unit, we note that families have long been viewed as the stable building blocks of society, playing a key role in nurturing, supporting, and socialising children [3]. However, major societal changes over past decades have been associated with changes to the longevity and diversity of family structures [4]. While most adults aspire to live in committed couple relationships and can expect their total married life to endure for an average of 32 years, the divorce rate now stands at 2.3 divorces per 1,000 population in Australia [5].

In 2011, 48% of divorces involved children under the age 18 years. This equates to approximately 50,000 children a year experiencing the divorce of their parents [6], with potentially serious negative long-term consequences for children [7]. Earlier meta-analyses reported that children of divorce had poorer academic achievement, conduct, psychological adjustment, and social development than children from intact families [7]. Recent studies have linked parental divorce with an increased risk of suicide attempts [8,9] and poor mental health [10].

Despite the heightened risk associated with parental divorce, it is important to note that the majority of children experiencing divorce do not experience long-term negative consequences [11], and researchers are increasingly interested in exploring the concept of resilience in relation to negative life experiences. Nevertheless, one documented risk factor for poorer outcome following family separation is ongoing or high-level parental conflict which has been associated with increased emotional instability, academic problems, behavioural and psychological stress, and uncertainty in children [12-14]. Conversely, the interests of children in separated families seem to be best served when parents are cooperative, child focussed, and reach formal agreement about their separation without litigation [12,15].

### Parental conflict

The process of separation is unique for each family, but commonly involves a period of heightened anger, anxiety and sadness, followed by positive readjustment after two to three years [16,17]. However, up to a third of separated couples are unable to settle their child-related issues [18], and for this subgroup, the conflict lasts longer and brings heightened risks for future well-being for themselves and their children [19]. High conflict associated with family breakdown also presents a serious challenge to achieving separation and child custody agreements as “primitive responses are triggered in parents poised to protect their children” [20]. Not only can conflict escalate into more serious forms of destructive behaviour, but can lead to parental disengagement and increased likelihood of negative outcomes for children, such as reduced cognitive competence [21].

The impact of marital conflict and hostility on children is increasingly understood as multi-dimensional, with particular aspects or styles of marital conflict accounting for specific adjustment problems in children [22,23]. Early studies found that important determinants of the effect of conflict on children included the form of the conflict (e.g. hitting, arguing, avoidance), the content of the conflict (e.g. sex, child rearing, money) and the amount of inter-parental conflict [21]. Substantial research has supported links between elements of marital conflict and specific patterns of marital communication, such as less facilitative and more aggressive behaviour [24]. Destructive communication, such as throwing insults or bringing up events from the past, breeds marital dissatisfaction in the face of life challenges [25], and is a strong predictor of divorce in the longer term [26]. Future research needs to include more differentiated measures of the types and severity of conflict to advance our understanding of the process and impacts of family breakdown, and how it can best be managed through services such as family mediation.

### Family violence

One area of particular concern is when parental conflict involves family violence, since the negative impacts of parental conflict are heightened when family violence is present. Domestic violence has emerged as one of the world’s most pressing issues, with the United Nations estimating that between 20% and 50% of all women worldwide have experienced physical violence at the hands of intimate partners or family members [27]. An estimated one in three Australian women have experienced family violence during their lifetime [28], making it a major health risk to women [29].

Children’s exposure to family violence or abusive behaviour is also of concern, with estimated rates varying from 10% to 50%, depending on samples and data source. For instance, a national US survey found that almost 10% of children saw one family member assault another [30]. In Australia, the 2005 Personal Safety Survey estimated that almost half of the men and women who had ever experienced violence by a current partner, had children in their care at the time [28]. As well, more than a quarter said that these children had witnessed the violence.

The definition and understanding of family violence has been subject to much debate [31,32]. Since the early 1990s, it has been acknowledged that single-factor explanations are inadequate and researchers have found it helpful to develop more differentiated typologies of family violence [15,31,33]. Recent amendments to the Family Law Act in Australia define family violence as a range of behaviours including: physical assault, harassment, emotional manipulation, financial abuse, and threatening behaviour [6]. Others have differentiated between various patterns of family violence [34]. For instance, Johnson identified four types: ‘common couple violence’ which arises from a specific incident and is not likely to escalate over time; ‘violent resistance’ refers to a woman fighting back at her aggressor; ‘mutual violent control’ where both partners are violent; and ‘intimate terrorism’ where the violence is part of a general pattern of control and is likely to escalate over time, is less likely to be mutual, and is more likely to result in serious injury.

Johnson also differentiated types of violence by the motivation of aggressors [34]. The most common form was situational violence involves partners reacting in the moment, but without an ongoing pattern of controlling behaviour. Another more serious form is an ongoing pattern of coercive controlling violence involving threats and intimidation and curtailment of personal freedoms and rights of the partner, what Johnson referred to as “intimate terrorism” [26]. Situational couple violence was found to account for 89% of violence in one survey, and coercive controlling violence accounted for 11% [35]. However, in relationships where intimate terrorism occurs, the violence has been found to occur more frequently and more severely than with situational violence [26].

Johnson’s work has been criticised because of its emphasis on physical violence and there is now increasing focus on differentiating among non-physical types of family violence such as psychological, emotional, social, and economic abuse [31,36]. Psychological abuse has been defined as: emotional/verbal abuse distinguished by devaluing or humiliating behaviours; and dominance/isolation which intends to produce compliance or conformity [31]. Economic or financial abuse is a form of non-physical violence and involves preventing a partner from knowing about or having access to family income. Non-physical forms of abuse are far more prevalent than physical abuse but there is increasing evidence that non-physical abuse can be a strong predictor of physical violence [37].

There is growing recognition of the need to also differentiate forms of violence by severity [31,37]. Moderate physical violence is described as serious but not life threatening behaviour such as pushing, grabbing, slapping, shoving. Severe physical violence includes threats using weapons, hitting with a fist or object [31]. This line of research is part of a growing call to move away from a simple present/absent approach to violence, and to take a more contextual view [36].

Gender is an important variable in any study of family violence. Generally, the most persistent and controlling forms of violence, such as ‘intimate terrorism’, are perpetrated by men, whereas more situational forms of violence are more likely to be instigated by both men and women [34,38]. However, gender differences are hotly debated. Despite strong evidence that most domestic violence is perpetrated by men against women, there are also now more than 100 empirical studies or reports suggesting that rates of domestic violence are equivalent. Therefore, future research on family violence needs to be gender-inclusive [39].

A number of methodological difficulties challenge our understanding of this social problem. Different studies have relied on different conceptualizations of aggression in families and use different sources of data, different age relationship status groups [40]. For example, studies of clinical samples tend to oversample severe forms of deliberate coercive male-driven violence, whereas studies using population-based samples or smaller studies of couples seeking marital therapy, may have downplayed the role of control by including many relationships characterized by violent arguments [39,41].

A key methodological issue is inconsistency in the conceptualization and measurement of couple conflict and family violence. The Conflict Tactics Scale (CTS) [42] has been widely used in research to measure the extent to which specific acts of violence have been enacted by both oneself and one’s partner, and to quantify severity as a sum of different types of violent acts. It comprises subscales measuring negotiation, psychological aggression, and physical assault, and distinguishes between minor and severe acts. The Conflict Tactics Scale Revised (CTS2) [32] included two additional scales (sexual coercion and injury), and some additional items in other subscales.

The CTS and CTS2 have been criticised on a number of levels, and researchers frequently modify it to meet the needs of particular studies [39,43-45]. At the very least, it has been argued that this scale should be supplemented with measures that tap additional aspects of violence and the contexts in which it occurs [26,43]. This is particularly the case to advance our understanding of the nature and impact of different forms of family violence within the family mediation context.

### Family violence and parental separation

Relationship breakdown and couple separation presents a critical period in the family violence cycle. Parental separation rarely means an end to violence since, for women in abusive relationships, the separation phase is the time of greatest risk of partner violence and homicide [46-48]. An analysis of Family Court of Australia cases found that violence was a factor in 75% of judicially determined cases [49]. In a later Australian Institute of Family Studies (AIFS) study of family violence allegations in the family courts, two thirds of separated mothers and over a half of separated fathers indicated that their child’s other parent had emotionally abused them before or during the separation [40]. The main forms of abuse were: physical violence (30%), threats (19%), emotional abuse (25%) and verbal abuse (25%). However, a key limitation of this research was its focus on simply counting acts of violence, highlighting a need for research that could assist the courts to better differentiate between types and severity of violence.

Another Australian study of separated couples shows that the majority of both males and females were frightened of their partner before, during, and after separation [12]. There was a distinct difference in the reasons why males and females said they were afraid of their partners’ behaviour. Females predominantly spoke about being afraid because of physical/sexual violence, and emotional/psychological abuse. Approximately half of the women spoke about being afraid because their partners would threaten their life, threaten them with and/or use weapons, and/or threatened they would commit suicide if abandoned. Male victims of violence did not report physical or sexual violence, but reported ongoing harassment and psychological abuse. Some of the men, while very distressed, ‘were not fearful of their former partner nor did they report feeling powerless’ [12]. Rivera and colleagues [50] argue that psychological abuse is not only more frequently experienced than other forms but can often be more painful and have longer lasting impacts, such as mental health and anxiety issues.

### Family violence and family mediation

Ongoing concerns about how the family law courts have dealt with cases involving family violence are well acknowledged [33,51]. This prompted policy changes requiring greater use of counselling, mediation, conciliation, and conferencing as the primary family dispute resolution process, rather than adversarial litigation through the courts [52].

Embodied in this Family Law Act was a list of children’s rights, including the child’s right to know and be cared for by both of their parents [51]. To facilitate parental cooperation in the ongoing joint care of children post-separation, the Australian Government created a national network of 65 community-based Family Relationship Centres (FRCs) which are intended to be “a first port of call” when families want information about relationship and separation issues [53]. FRCs provide information and referral services to families and free mediation services to separating couples. Attendance at family mediation prior to initiating court proceedings became mandatory for most separating parents who are obliged to make “genuine effort” to resolve their issues. Clients affected by family violence are potentially exempt from Family Dispute Resolution (FDR) services and FDR practitioners are obliged to screen for the presence of high conflict, coercion and violent behaviour and refer those clients deemed inappropriate directly to court.

Implementation of family mediation policies in the context of family violence is nevertheless fraught. Research suggests that family violence is not always recognised by mediation practitioners [51,54], and that even when it is recognised, appropriate actions aimed at creating or preserving safety are not always taken [55]. Furthermore, practitioners express concern about the high percentage of families presenting with disclosed problems of family violence [12]. For instance, 90% of couples attending divorce mediation reported partner violence in one study and only about 7% of cases were actually screened out of mediation [56].

A few studies provide conflicting findings: family violence was adequately screened and monitored in one study [57], and separated couples can achieve comparable levels of agreement, despite different violence histories [58]. However, this is not so true for serious violence. Only 15% of the more violent couples reached full agreement in mediation, compared with overall agreement rate of 55% in another study, suggesting that the most violent couples effectively self –select themselves out of the mediation process [59].

In summary, there have been calls for better research on the processes of separation and divorce in the context of high conflict and family violence [60,61]. This study focuses specifically on couples attending family mediation as a mandatory step in resolving disputes prior to court attendance. It addresses a gap in the research literature by assessing the types and severity of multiple forms of family violence among a high risk sample of couples attending family mediation centres and exploring relationships between types of abuse. We use previously validated scales and additional scales to tap broader definitions of partner abuse. We also examine mediation outcomes and the impact of different types of violence.

Such research is important since much of the violence seen within health and human services is understood to fall into the category of intimate terrorism, involving an escalating pattern of coercive control [34]. These are the sort of cases which, under Australian Family Law, should find their way directly into the court system, since they are generally not cases that can or should be assisted by mediation [17]. Yet, there is little research currently that casts light on the type, extent or severity of violence in couples presenting at family mediation, and how that influences mediation or referral to the courts.

### Study aims

The primary aim of this study is to examine family violence in the context of parental relationship breakdown and its impact on the family mediation process and post‒separation decision making. More specifically, the aims are:

1. To map the profiles of clients seeking family mediation in terms of their relationship, mental health and quality of life indicators, history of violence, and socio-demographic factors.

2. To determine the existence and prevalence of family violence within the relationship and how this influences mediation processes and mediation agreements and other decision that parents make in relation to post-separation parenting arrangements.

3. To evaluate clients’ satisfaction with mediated agreements and to determine which factors influences clients’ satisfaction at pre-mediation, post-mediation and 6 month follow-up periods. Potential factors include: characteristics of pre-test group (demographics, relationship history, health and well-being); mediation processes (experience of mediator, number of sessions); and client assessment of mediation service (e.g. satisfaction with the outcome of mediation, reduction in conflict).

4. To evaluate the durability of mediated agreements, particularly parenting agreements at 6 month follow-up.

## Methods

### Study design

The study used a mixed method longitudinal design to evaluate the impact of family violence on family mediation process and outcomes among clients presenting at nine family mediation centres across metropolitan, outer suburbs, and regional/rural sites in Victoria, Australia. The longitudinal design involved data collection at pre-test, post-test on completion of mediation, and a follow-up post-test six months after completion of mediation. Quantitative data were collected using self-administered surveys at the three time points. Qualitative data were also collected via a telephone interview at the final post-test period. All consenting participants who attended mediation were included in the study.

### Setting

The setting for this study was the network of nine family mediation centres or Family Dispute Resolution providers operated by Relationships Australia Victoria (RAV). Five sites were located in the Melbourne metropolitan area, one in Melbourne’s outer suburbs, and three in regional and rural centres of Victoria.

Relationships Australia is an approved family mediation service provider under the *Family Law Act* 1975. It operates as an incorporated, not-for-profit community-based secular organisation that has been delivering services in Victoria since 1948. It provides services to the community to build stronger relationships which enhance the lives of individuals, families and communities. As the largest provider of both family mediation and family violence prevention programs within Victoria, and the largest provider of family mediation in Australia, it is in a unique position to support and promote good quality clinical research and strengthens the applicability of our findings.

### Sample

Our sampling frame includes all adults referred to the nine family mediation services over a nine month period in 2011. We aimed to recruit at least one individual from each couple. Given high levels of conflict in this sample, we did not consider it feasible to recruit couples as the primary sampling unit.

### Recruitment and procedures

When potential participants attended an individual or group information session, the mediator informed them about the study and clients were invited to take an envelope containing relevant information and the pre-mediation questionnaire and pre-paid envelope. If participants chose to be part of the study, they completed the questionnaire and consent form and returned them to the research team. Participants then received a post-mediation questionnaire on completion of their mediation. A longer-term post mediation follow up telephone interview was conducted at six months post-test. A monetary incentive ($25) to complete the surveys/interview was offered at each stage of the research. Mediators completed a post-mediation survey following completion of the mediation to provide their assessment of the dispute, mediation process and outcome.

To facilitate the process of recruitment, a research assistant regularly visited each participating RAV site to collect forms, review recruitment processes and handle any difficulties experienced by agency staff. Mediation staff were contacted regularly to record when participants’ files were closed so that they could be contacted for the post mediation follow up. This process commenced in March 2011 and continued for approximately 9 months until a suitable sample size of 121 participants was achieved.

Participants who decided not to proceed with mediation or decided to drop out before the completion of mediation were still invited to complete the post and post- post mediation questionnaire which would give the study a quasi-control group to compare with those who proceeded and completed their mediation sessions.

The project was approved by La Trobe Human Research Ethics Committee and RAV’s Research and Ethics Committee.

### Measures

Pre-mediation and post mediation self-report questionnaires were mailed out to participants. The 6 month post mediation follow-up involved a telephone interview and a mailed self-report questionnaire. Table 1 provides a summary of the research aims, methods of addressing these and potential benefits.

**Table 1:** Research aims, methods of addressing these and potential benefits

**Research aims**	**Methods**	**Benefits**
1. To map the profiles of clients seeking family mediation in terms of their relationship, mental health, history of violence, and socio-demographic factors	Baseline survey. Measurement of relationship variables including level of couple conflict and family violence (CTS2, financial abuse, controlling behaviour, domination and intimidation), level of acrimony in the relationship and parenting alliance. Other variables include psychological wellbeing, and demographic features	To increase knowledge of the demographic and relationship profile of separating adults who attend family mediation, the prevalence of family violence and conflict, and their capacity to negotiate parenting issues. Factors associated with levels and types of family violence will be assessed
2. To determine the existence and prevalence of family violence within the relationship		
		The measurement of violence used in this study addressed some of the limitations of current scales by measuring a wider range of abuse domains, such as financial abuse, controlling and coercive behaviour. The study will enhance understanding of family violence within the post-separation and mediation context
3. How does the presence of family violence influence mediation processes and mediation agreements and other decision that parents make in relation to post-separation parenting arrangements	Assessment of parties’ experiences of mediation, mediators assessment of parties’ level of conflict and their ability to mediate successfully, and what outcomes were achieved in terms of parenting agreements	The study explored how mediators screened for violence, whether they referred on, how they handled violence during mediation sessions, and the decisions that were made about parenting agreements in the light of the couple history and experience of violence
4. To evaluate clients’ satisfaction with mediated agreements and determine predictors of clients’ satisfaction post-mediation and 6 month later. Potential predictors include: pre-test characteristics, and mediation processes (experience of mediator, number of sessions)	Measurement of presence of type and severity of violence before, after and at 6 months after mediation to assess any changes	This longitudinal survey allows us to track changes in violence and acrimony between separated couples after mediation and whether mediation was able to positively influence this behaviour
	Measurement of changes to parental conflict before and after mediation and at and 6 months.	Knowledge about the parties experience of the mediation and their feedback about the process and outcomes can better inform clinicians about their needs
5. To determine durability of mediated agreements, eg, parenting agreements at 6 month follow-up	The qualitative survey at post-post mediation assessed the strength of the parenting agreement	The study examines how parents fared up to 6 months after mediation, to assess whether mediated agreements lasted, or whether alternative plans developed. It also examines predictors of durable and successful agreements and impact on parenting roles

#### Pre-mediation questionnaire

The pre-mediation questionnaire contained a total of 101 questions divided into four sets of items: socio-demographics, mental health, relationship, and conflict variables. In the post-mediation and post-post mediation questionnaires most demographic questions were omitted and additional items about their experience of the service and outcomes were included.

##### Socio-demographics

This section contained 9 items that asked participants for their demographic information (birth date; gender; country of birth, years in Australia if born overseas) and nature of their relationship with the person they are mediating with. These latter questions asked about: whether they were still living together; number of years they have been in a relationship; if separated, for how long; and the number of children from this relationship. Participants also indicated their relationship status (married, de facto, de facto separated, separated but not divorced, or divorced).

##### Psychological distress

Level of psychological distress over the previous four weeks was assessed using the *Kessler 6*, a widely used screening scale and treatment outcome measure [62,63]. The six questions ask about depressive and anxiety symptoms, such as feeling hopeless or nervous, in the last 4 week period. Participants rated how often they experienced the symptoms on a five point scale ranging from ‘all of the time’ to ‘none of the time’, high score indicating good psychological health.

##### Relationship conflict

The nature of the parental relationship post separation was assessed using a 5 item scale developed from Emery’s Acrimony Scale [64]. *The Acrimony Scale (AS*) is a 25-item measure of co-parenting conflict between separated or divorced parents that yields a single acrimony score, the mean of all items, with higher scores indicating greater conflict and more co-parenting difficulties. The 5 items were selected by an in-house factor analysis of AS scores from a large sample of 800 mediation clients which found that over 95% of variance was accounted for by these 5 items. The 5 items referred to hostility, anger and whether these feelings were expressed in front of the children. Items were rated on a 5 point scale, 1 = almost never, 4 = almost always, and 5 = not applicable to your situation.

##### Parental alliance

The strength of the parenting alliance between the couple was assessed using the 20 item *Parental Alliance Inventory (PAI)* developed by Abidin and Brunner [21]. Parenting alliance is a central variable influencing parenting behaviour and has been related to children’s adjustment. Abidin and Brunner [21] reported a Cronbach’s alpha of .97 for the PAI, and established moderate concurrent and construct validity by correlating the scale with measures of marital satisfaction, parenting stress and parenting style. All items were reworded to refer to children in the plural, where Abidin and Brunner [21] used wording that referred to ‘child’ in the singular. The scale asked participants to rate on a 5 point Likert scale how strongly they agreed with statements such as “my children’s other parent and I are a good team” and “I believe my children’s other parent to be a good parent”. Lower scores indicating a greater parenting alliance, with ‘1 = strongly agree’ and ‘5 = strongly disagree’.

##### Conflict

The presence, nature and severity of family violence was measured by four scales.

*Conflict Tactics Scales Revised* (CTS2) [32] was used to measure the extent to which partners engage in psychological and physical attacks on each other as well as their use of reasoning or negotiation to deal with conflicts. The earlier version CTS [42,65] has been the most widely used measure of intimate partner violence, with known validity and reliability [66]. The updated CTS2 [32] provides a better operationalization of the distinction between minor and severe acts and is useful in that it asks the client to report on his or her own behaviour in addition to their ex partner’s behaviour. Although the CTS has received criticism for being insufficiently sensitive to circumstances and context [40], it is widely used and across different samples [44,67]. Concurrent validity [68] and construct validity have also been well established, with the CTS correlating with many different measures of etiology and impact of partner violence (for review, see work by Straus and Mickey [67]. It is often employed as a test of concurrent validity when similar measures are developed [69].

The CTS2 scale included 32 items from four subscales: Negotiation (6 items), Psychological Aggression (8 items), Physical Assault (12 items), and Injury (6 items). Participants were asked to rate on an 8 point Likert scale how often they and their former partner enacted the behaviours in the past year, with a range of ‘0 = this never happens’ to “7 = this happens more than 20 times in the past year’. The scale and its subscales have been found to have reliable internal consistency, with alpha’s ranging between acceptable (.68) to excellent (.95) [32,44,67,70,71]. It has also been found to have good test-retest reliability in a sample of court-mandated men in a batterer intervention program [44]. The sexual violence subscale was omitted due to sensitivity of the topic in a family mediation context, low internal consistencies [71], and low test-retest reliability [44].

There has been some debate about the factor structure of the CTS2, and the number of factors varies between 3, 4 and 5 factor models, depending on which scales are included or excluded from analyses [32,70-72]. As the scale has been validated in its original factor structure, the present study chose to use Straus’ original subscales with the exception of the sexual coercion scale, thus conceptualising the CTS2 as a 4-factor structure of negotiation, psychological aggression, physical assault, and injury.

##### Controlling and intimidating behaviour

In order to tap the areas of emotional abuse and controlling behaviour that are not directly addressed by the CTS2, a scale was developed to measure controlling and intimidating behaviour. The items were modified from previous measures which had examined emotional abuse [66] and controlling behaviours [43]. The seven item scale tapped behaviours that are controlling and jealous in nature, such as ‘one partner kept the other from seeing friends’ and ‘one partner falsely accused the other partner of having an affair’. As with the CTS2, participants are asked how often they and their partner had enacted this behaviour on an 8 point scale which ranged from ‘0 = this never happens’ to “7 = this happens more than 20 times in the past year’. The scale had good reliability with a Cronbach’s alpha of 0.82.

##### Financial abuse

A four-item scale was developed to measure *financial or economic abuse* since this is recognised as an important aspect of dominance/isolation behavior by limiting a partner’s access to money [31]. The created scale included a modified item from the Controlling Behaviour Index [43]: reworded as ‘My partner deliberately kept me short of money’. The other three items were developed by the research group to measure financial domination described as a way to limit a partner’s access to money and other resources in order to produce compliance or conformity [37]. The items asked participants to rate how often they and their partner excluded the other from financial decisions, kept financial situations secret, and controlled the money in the relationship. The four items were measured with an 8 point Likert scale, which ranged from ‘0 = this never happens’ to “7 = this happens more than 20 times in the past year’. This scale also had good reliability with an alpha of 0.85.

##### Screen for family violence

The 16-item *Family Dispute Resolution checklist* was used routinely by RAV staff to screen for violence and to assess participants’ capacity to mediate without fear and intimidation from their ex-partner, and was thus included in this study. Participants rated how much they agreed with each statement on a five point Likert scale, ranging from ‘1 = strongly agree’ to ‘5 = strongly disagree’, higher score indicating greater risk. Some examples of the items include “my partner was aggressive and dominating in our relationship”, “I feel dominated/intimidated by my former partner” and “I was afraid of my partner”.

### Post-mediation questionnaire

The post-test mailout survey contained 85 questions. The Kessler Health scale, AS and PAI were re-administered to assess any changes to the FDR process and outcomes. The remaining questions measured various aspects of the mediation process and outcome as well as assessing the participants’ experience of and satisfaction with the sessions.

#### Mediation processes

Six items asked about the focus of mediation, such as property or parenting issues, and about the outcomes. In addition, satisfaction with the information/intake session and the mediation was scored on a 5 point Likert scale, with higher scores denoting that the sessions were more helpful. This section also asked about the possible longer term benefits of mediation, even if an agreement was not facilitated. The six questions were modified from a previous evaluation of the Broadmeadows Family Relationships Centre (BFRC), which looked at client satisfaction and specific outcomes of the mediation [73].

#### Client assessment of mediation (CAMS)

Twenty items from 10 scales of the Client Assessment Mediation Services questionnaire (CAMS) [74] were included and participants were asked to score how strongly they agreed with a variety of statements on a 7 point Likert scale, which ranged from 1 = very strongly disagree to 7 = very strongly agree. The items were taken from the following subscales: Effective/Sensitive Mediator (e.g. ‘I felt that the mediator too often favoured my (former) partner’s point of view’ (1 item); Empowerment (e.g. ‘As the result of mediation, I am more confident about my ability to stand up for myself’) (3 items); Impartiality (e.g. ‘The mediator(s) seemed quite impartial when it came to resolving differences between me and my (former) partner’ (4 items); Focus on Issues (1 item); Impact on Spousal Relationship (e.g. ‘I now believe that I can resolve any future disagreements with my (former) spouse without outside help’ (3 items); Satisfaction with agreements (2 items); Emotional satisfaction (1 item); and Adequacy of Information (e.g. ‘I feel that I received enough information to protect my own best interests during the mediation’) (2 items).

#### Children and mediation

Six questions asked about client’s satisfaction with outcomes relating to children, parenting, and parenting agreements: three items from the CAMS; three from the BFRC questionnaire. Items included statements such as ‘The mediators provided useful information about parenting during the mediation session’ [73] and “Mediation helped to identify useful ways to arrange our parenting responsibilities” [74], and responses were on a 7 point Likert scale.

A further two questions asked parents to rate their satisfaction with discussions and outcomes regarding the children on a 5 point likert scale which ranged from 1 = not at all satisfied, to 5 = very satisfied. Four open ended questions about the parenting agreements were also included enabling participants to offer more detail. Finally, one question asked whether the parenting agreement was still working (response options being yes, no, and partially). The latter seven questions were drawn from Balvin and colleagues [73].

#### Conflict and mediation

The final set of 11 questions were modified from the FDR and a were a mixture of closed questions and open-ended questions where participants could provide a more detailed response about their experiences of safety in mediation and the safety of the parenting agreement developed for their child(ren). They included participants’ experiences of feeling dominated, intimidated and fearful during mediation; information about the existence of an intervention/apprehended/domestic violence/restraining order, whether there was a breach of the order and if the participant or child(ren) had experienced abuse outside the mediation session. Finally, participants were asked about any referrals that mediators may have suggested or provided.

### Mediator questionnaire

This measure contained 16 routinely collected questions, modified from CAMS. The mediator was asked whether the case progressed to FDR and the reasons why it did not proceed. Whether cases did or did not proceed, mediators were asked for each case about their perception of the parenting alliance, level of inter-parental conflict, whether family violence was a current or past issue, and type and perpetrator of violence. Mediators were also asked whether there was an intervention order, whether they issued a certificate so that the ex-partners can terminate mediation and can access the Family Court and what effect family violence, if present, had on participation in mediation. Referral information was also requested.

Where cases proceeded to FDR, data routinely collected by the mediator was also used: model used in mediation, hours in mediation, level of agreement, ages of the children involved, and their level of confidence regarding the implementation of the agreement developed in mediation, ranging from 1 = not at all confident, to 5 = very confident. Apart from assessment of the success of parenting agreements, this information is routinely recorded by the mediator as part of RAV’s administrative processes.

### Post-post mediation questionnaire

Three scales from the pre and post questionnaires were re-administered in the 6 month followup mailout questionnaire: Kessler 6 [62], AS [64], and PAI [21].

The phone interview consisted of 63 questions in nine sections to explore 6-month outcomes of mediation, particularly related to parenting arrangements and parental relationship.

#### Relationship with former partner

Respondents were asked whether they were still separated from their partners, the main reasons for the separation, and whether they had felt intimidated or fearful in the presence of their ex-partner.

#### Parenting and children

Open-ended questions asked about the pre-separation distribution of childcare, their understanding of “shared parenting”, current care arrangements, experiences of post-separation parenting, and whether parenting was easier, harder or the same as pre-separation.

#### Parental conflict post separation

Respondents were asked about any post separation conflict between ex-partners in terms of triggers, frequency, severity, setting, and impact on children.

#### Parenting agreement

Participants were asked about the Parenting Agreement, including issues discussed (resolved and not resolved), reasons for failure to resolve, any changes to original agreement, and any difficulties with implementation. Using a 5 point Likert Scale, where 1 = not at all and 5 = strongly agree, respondents were also asked to rate 11 influences on the parenting agreement, both positive and negative, such as, ‘Information and guidance from the mediator or family dispute resolution practitioner helped assist with the parenting decisions for our children’.

#### Post parenting agreement

Respondents were asked how much they agreed with seven statements about their children’s perception of the parenting agreement, responding on a 5 point Likert Scale (1 = strongly agree and 5 = strongly disagree). Participants were also invited to comment further on these items. Four additional open-ended questions included: Issues around safety, whether the mediator had acted on safety concerns if relevant, satisfaction, and flexibility of the arrangements. If no agreement had been reached, the respondent was asked to comment on their experience.

#### Mediation

Questions asked about the pathway to mediation, time frames, the process of completing an agreement, what they would do differently given their current knowledge, and the value of mediation.

#### Use of other services

Respondents were asked yes/no questions about whether they had accessed a number of different services (e.g. counselling, refuge, independent legal advice). If no service accessed, participants were asked if they thought it would have been useful to do so (response options = useful/not useful).

#### Family violence

Family violence issues were explored within the mediation context. For example, respondents were asked to talk about their experience in mediation and whether the agreement ameliorated the level of violence post separation. In the final section, respondents living with family violence were asked to provide feedback about ways to improve services. Themes will be derived using thematic analysis.

#### Mediation service

Finally participants were asked for any further comments on mediation.

### Ethics

Ethical approval for the study was obtained from the La Trobe University Human Research Ethics Committee and the Relationships Australia Victoria Ethics Committee.

### Data analysis

Descriptive analyses will be conducted to map the profile of participants referred to family mediation services, in terms of demographic and relationship characteristics, the types and severity of conflict and abusive behaviour, parenting alliance and wellbeing. Comparisons will be made by gender, and between those who proceed with mediation versus those who do not. Structural equation modelling will be used to explore the relationships between variables. The outcomes of family mediation will be examined using regression models. All statistical analyses will be undertaken using SPSS-19. The qualitative interview questions will be coded using thematic analysis.

#### Power analysis

For the long-term evaluation, a power analysis was conducted as follows. The estimated client base for the nine participating family mediation sites was approximately 600 new couples (1200 individuals) per year. We conservatively estimated that at least one member of 30% of couples (N = 180) would consent to participate in the study, and that an estimated 10% of those were likely to be declared ineligible following the initial session, yielding approximately 162 eligible and consenting individuals at baseline. We anticipated a 60% attrition rate by post-mediation follow-up (n = 97) and a further 30% by 6 month follow-up, yielding a final evaluation sample of 68 participants. This is sufficient to predict change across time for key outcomes, with a medium effect size, 80% power and a .05 significance level.

## Discussion

The coinciding of legislative reforms and sector concerns regarding the prevalence and impact of non-physical forms of family violence warrants an examination of the family violence profile of FDR clients. If we accept that there is a wide definition of family violence events that require a corresponding range of interventions, then parents capacity to participate in FDR will vary as well [45,75]. This paper examines this aspect of family mediation through measuring the profile of violence being experienced by parties presenting at family mediation services and the consequent impact of this profile on the FDR process, the immediate outcomes of FDR and the subsequent durability and sustainability of these outcomes.

Although there has been a number of major studies which as looked at the incidence of family violence in separated couples, they have mainly looked at cases that present at the Family Court or as part of large scale surveys of the general population. The significance of this study lies in mapping of client profiles and their exposure to violence during their relationship, comparing the characteristics of this violence and how it manifested in mediation and what kinds of parenting agreements they were able to establish and how well they endured over time. It also evaluated client satisfaction with the service providers and the outcomes that they mediated.

The study will also provide an important contribution to the evidence base in determining better screening measures in FDR. Having a better understanding of participant profiles will allow for more targeted screening processes as well as guiding decisions about when mediation could be success with some types of family violence. Other strengths of the study include the large sample size compared to previous studies of this population and the longitudinal design of the study which offered the opportunity to gather qualitative data which will enable further exploration of participants’ experiences and clarification of the major themes emerging from the study outcomes.

## Abbreviations

FDR: Family dispute resolution; FRC: Family relationship centre; CTS: Conflict tactics scale; CTS2: Conflict tactics scale 2; AIFS: Australian Institute of Family Studies; DV: Domestic violence; RAV: Relationships Australia Victoria; FDRSPs: Family dispute resolution providers; AS: Acrimony scale; PAI: Parental alliance inventory; BFRC: Broadmeadows family relationship centre; CAMS: Client assessment mediation services; GLAMM: Generalized linear latent and mixed model.

## Competing interests

The third author is currently CEO of Relationships Australia Victoria, the setting for the study. Relationships Australia Victoria provided funding as an Australian Research Council Linkage Grant partner in the project. While he was actively involved in the design of study and facilitating access to the setting, there was no inappropriate influence on the design or conduct of the study. There are no other competing interests.

## Authors’ contributions

HC had primary oversight of the project and contributed significantly to design and methodology, supervision of staff, and writing of study protocol. MS contributed significantly to design of the study and writing of study protocol. AB (CEO of Relationships Australia Victoria) contributed to study design and manuscript preparation, facilitated access to relationships services and recruitment processes. All authors read and approved the final manuscript.

## Pre-publication history

The pre-publication history for this paper can be accessed here:

http://www.biomedcentral.com/1471-2458/14/57/prepub
